# Can I Say “No”? How Power Dynamics Hinder Consent in University Settings

**DOI:** 10.1177/10778012251338485

**Published:** 2025-05-05

**Authors:** Manon Bergeron, Ihssane Fethi, Marie-Andrée Pelland, Lise Savoie, Karine Baril, Jacinthe Dion, Marie-Hélène Ouellette, Geneviève Paquette, Tarah Paul, Anne-Sophie Ponsot, Sandrine Ricci, Cindy Viau

**Affiliations:** 1 229169 Université du Québec à Montréal, Montréal, QC, Canada; 2 5622 Université de Montréal, Montréal, Québec, Canada; 3 5568 Université de Moncton, Moncton, New-Brunswick, Canada; 4 59310 Université du Québec en Outaouais, Gatineau, QC, Canada; 5 Université du Québec à Chicoutimi, Chicoutimi, QC, Canada; 6 Centre d’aide et de lutte contre les agressions à caractère sexuel (CALACS), St-Agathe, QC, Canada; 7 248217 Université de Sherbrooke, Sherbrooke, QC, Canada; 8 Conseil québécois LGBT, Montréal, QC, Canada; 9 Groupe d’aide et d’information sur le harcèlement sexuel au travail, Montréal, QC, Canada

**Keywords:** sexual consent, sexual violence, sexual harassment, university, power dynamics

## Abstract

Teaching assertive sexual consent has been emphasized to combat gender-based and sexual violence in higher education (GBSVHE). However, the relationship between sexual consent and GBSVHE is complex, and teaching “say no” seems insufficient to eradicate these issues. This article examines 52 accounts of individuals who were victims of GBSVHE. The qualitative analysis revealed that power dynamics significantly hindered the acknowledgment of nonconsent in university settings, especially through gendered dynamics, social norms, and authority relationships. These findings support the need to raise awareness of the influence of power dynamics, foster ethical conduct, and promote respectful and egalitarian relationships.

Recent years have seen an upsurge in movements to denounce the pervasiveness of gender-based and sexual violence in higher education (GBSVHE). These movements condemn the prevailing atmosphere of tolerance, trivialization, and normalization of GBSVHE and call to dismantle the structural dimensions that create, maintain, and perpetuate such violence (Ricci & Bergeron, 2019). Several initiatives have been implemented in universities to prevent and address GBSVHE, including affirmative consent training (Burton et al., 2021; Kubota & Nakazawa, 2022). However, the relationship between consent and GBSVHE is complex, and several authors argue that simply teaching “say no” is insufficient to eradicate sexual violence (Morin et al., 2019; Pugh & Becker, 2018; Savoie et al., 2019).

## Gender-Based and Sexual Violence in Higher Education

Gender-based violence (GBV), including physical, psychological, and sexual violence, refers to harmful acts directed at a person because of their gender (SV). Both GBV and SV are rooted in gender inequality and unequal power dynamics, with GBV encompassing acts that are not necessarily sexual in nature. We use the term GBSVHE to address such violence occurring within higher education settings. In addition to its high prevalence, GBSVHE is likely to induce consequences that can significantly disrupt daily functioning (Bergeron et al., 2016). Victims are more likely to report repercussions on their mental health (e.g., depression, anxiety, post-traumatic stress symptoms), changes in their lifestyle, and difficulties in pursuing academic or professional activities at their university.

This article adopts a feminist conceptualization of sexual violence, viewed as a continuum that includes acts of ordinary and normalized violence, sexual harassment, and sexual assault (Kelly, 1987; Savoie et al., 2018). This perspective allows us to consider the structural dimensions of such violence, seen as a manifestation of systems of oppression (e.g., sexism) and a culture that trivializes, normalizes, and tolerates them. Focusing on social and contextual dimensions allows us to understand how these types of violence are the product of the social organization rather than the actions of a handful of deviant individuals or isolated cases. This approach also expands the avenues for explanation and intervention.

Sexual violence within the university microcosm reflects the broader social organization. Universities are environments where interactions between groups of individuals are structurally hierarchical and where dynamics of power and domination mirror those in contemporary societies. Moreover, several characteristics of the university setting can foster GBSVHE: a climate of tolerance and discrimination toward certain social groups, precarious working or learning conditions, activities centered around (excessive) alcohol consumption (e.g., fraternities, orientation week), and a hegemonic and heteronormative culture of masculinity (O'Connor et al., 2021; Tashkandi et al., 2022). Therefore, it is critical to consider these factors when considering the notion of consent to develop a better understanding of and response to the issues surrounding GBSVHE.

## The Complexity of Sexual Consent: Willingness or Consent?

Contrary to the simplicity of popular slogans such as “no means no,” consent is a complex concept, as emphasized by several authors (Beres, 2022; Savoie et al., 2019). Muehlenhard et al. (2016) define consent as a “discrete event” that encompasses both the relationship with the other individual and the relationship with oneself. They describe it as an “internal state of willingness,” meaning that consent is not always directly observable and must be assessed through verbal and nonverbal cues, as well as interpretations of an individual's internal state and willingness to engage in sexual activity (p. 462). Similarly, according to Beres (2007), sexual consent is primarily conveyed through the body, emotions, or facial expressions.

Several individual, relational, and contextual aspects can influence sexual consent, including understanding the other individual's consent and having the autonomy and ability to give, withhold, or withdraw consent (Kubota & Nakazawa, 2022; Muehlenhard et al., 2016; Willis et al., 2019). Factors such as gender, age, sexual orientation, gender stereotypes, beliefs about consent, the presence of violence, and power dynamics, among others, can also determine the degree of freedom an individual has to exert sexual consent. Consent also depends on several contextual elements, including the nature of the relationship (e.g., casual or committed relationship) and the state of consciousness (e.g., alcohol consumption, mental state).

Several studies suggest that consent extends beyond a simple “yes” or “no” response to sexual activity. Navigating the scene of sexual relations during their university years can lead to ambivalent situations as students report that their sexual consent is sometimes given despite internally not wanting the sexual activity (Beres, 2022; Hirsh & Khan, 2020; Morin et al., 2019). This is one of the reasons why there have been multiple sexual violence prevention initiatives promoting enthusiastic consent or encouraging affirmative consent (Burton et al., 2021; Kubota & Nakazawa, 2022). However, addressing GBSVHE solely from the perspective of consent communication has its limitations, as even situations that meet the legal standard for affirmative consent may not reflect genuine desire or willingness.

## Ambiguity and Miscommunication of Consent?

Beres (2022) explored the question of consent communication and identified that it is often characterized as ambiguous in research. Several authors argue that using this notion of ambiguity can inadvertently legitimize attempts to continue the sexual activity, potentially leading to sexually abusive behaviors (Beres, 2010; Pugh & Becker, 2018; Savoie et al., 2019; Willis et al., 2019). Such situations do not reflect a misunderstanding but a sophisticated comprehension of consent communication and social norms (Hansen et al., 2010). Miscommunication can be used to justify the overstepping or even transgression of the intimate boundaries of the individual expressing “no” (Beres, 2022; Jeffrey & Barata, 2019). Kubota and Nakazawa (2022) summarize these issues as follows:

Sexual consent is ambivalent: if we acknowledge its ambiguity and complexity, then sexual violence may be justified through miscommunication theory; if we claim that it is simple, then we fail to consider the divergent states of internal/external consent and the existence of various influencing factors. (p. 16).

This ambivalence can be overcome by promoting ethical conduct in sexual behavior. In this sense, Hirsch and Khan (2020) introduce the idea of sexual citizenship to prevent situations perceived as ambivalent, advocating for students to respect their partners’ sexual desires and projects. Similarly, Beres (2022) adopts the term “tuning in” to describe how consent communication must be understood through “sexual ethics.” Sexual ethics involves grasping subtle expressions in interactions, including nonverbal cues when one is mindful of their partner's willingness and wants. Beres (2022) emphasized that the focus should not be solely on verbal consent but on educating about sexual ethics:

It includes a complex, empathetic reading of sexual partners to create a mutual and connected experience. This reading is not based on an assessment of specific behaviors, but instead recognizes subtle shifts in tone, body tension or relaxation, invitations and expressions. Also, the notion of “being present” was also palpable with participants reporting that they can read it in others and that their partners also can accurately read them. (p. 150)

From this perspective, sexual assault cannot be understood as miscommunication but as unethical conduct that does not respect the desires and wants of the other. Thus, comprehensive and nuanced consent education is needed to address these complexities and promote ethical sexual behavior. Nuanced consent education involves moving beyond a “yes” or “no” binary as ideal standards for consent and navigating the complexities of consent as advocated by several scholars and emphasized by gray area trainings (Beres, 2014; Setty, 2023).

## Negotiating Consent Within Higher Education

Institutions play a crucial role in shaping the understanding and practice of consent within their environments. Universities, as institutions, have significant power to influence behaviors and attitudes through their policies, culture, and responses to sexual violence. Previous work has highlighted how individual behaviors, peer dynamics, and institutional policies interact to create environments where sexual assault is more likely to occur (Hirsch & Khan, 2020). Thus, understanding consent within higher education requires examining how institutional norms, systems, and practices influence individuals’ experiences with sexual violence and consent.

Such an examination is critical, considering institutional betrayal, which occurs when institutions fail to prevent or respond to sexual violence, thus perpetuating harm (Smith & Freyd, 2014). Institutional betrayal happens when universities overlook helping victims of sexual violence, shift blame onto victims rather than aggressors, or neglect to safeguard survivors despite claiming to address sexual violence. Thus, efforts to eradicate GBSVHE require changes at the individual level as well as reforming institutional norms and practices.

## Limitations of Current Knowledge

Research on sexual consent in higher education has been conducted almost exclusively with undergraduate students (Burton et al., 2021; Kubota & Nakazawa, 2022; Morin et al., 2019) and has not explored the experiences of other groups in the university population (e.g., graduate students, faculty, and staff members). Thus, the current state of knowledge could benefit from an exploration of the experiences of other groups within the university setting. Previous studies have also primarily focused on short- or long-term heterosexual relationships (Fenner, 2017; Hirsh & Khan, 2020). This research has, for example, identified the weight of heteronormative norms that position women as the gatekeepers of consent and men as the initiators of sexual activity and has resulted in various recommendations for addressing and preventing GBSVHE (Fenner, 2017; Muehlenhard et al., 2016; Wood et al., 2019). However, several authors have noted a disconnect between some of these recommendations and the actual experiences of young adults (Burton et al., 2021; Muehlenhard et al., 2016; Wood et al., 2019). Thus, developing a more grounded understanding based on individuals’ experiences could lead to educational approaches that are closer to their reality.

In addition, research has overlooked the influence of the university setting and would benefit from identifying situational or interpersonal factors that may restrict individual freedom (Muehlenhard et al., 2016; Wood et al., 2019). In certain situations, the freedom of individuals to consent or refuse is limited by their circumstances, and it is critical to explore the constraints that are at play within the university setting. Such research would allow us to understand how coercion is exercised and thus go beyond a focus on the presence or absence of consent.

In response to the various gaps identified in the scientific literature, this article examines the notion of sexual consent as it appears in the accounts of individuals who have been victims of GBSVHE. The purpose of this study is (1) to explore how the university population, including students and employees (e.g., faculty, staff), refers to consent in their accounts and (2) to explore the influence of university settings, events and norms (e.g., party contexts, gender norms, power dynamics) on how the notion of consent is articulated. By understanding how consent is being negotiated in the shadow of institutional norms, this study seeks to provide insights into improving educational programs to address GBSVHE.

## Method

The study is based on data collected in the context of the Study on Sexuality, Security, and Interactions on University Settings, which aimed to document situations of GBSVHE experienced by individuals studying or working at six universities in Quebec, Canada (Bergeron et al., 2016). The data was collected between January and May 2016 via online solicitation, posters, and institutional email lists. To assess experiences of GBSVHE, a French adaptation of the Sexual Experiences Questionnaire (Fitzgerald et al., 1999) was used. The questionnaire consists of 21 items and distinguishes three forms of sexual violence: *sexual harassment* (verbal and nonverbal behaviors that are not aimed at sexual cooperation and result in insulting, hostile, and degrading attitudes), *unwanted sexual behaviors* (offensive, unwanted, and nonreciprocal verbal and nonverbal behaviors that include attempted rape and sexual assault), and *sexual coercion* (blackmail against future considerations related to employment or the learning environment). For each of the 21 items, participants indicated whether someone affiliated with the same university had committed the act against them. When an individual reported experiencing at least one of these acts, an additional section invited them to respond to an open-ended question that asked them to describe a GBSVHE situation they experienced:

This section allows you to describe this event in greater detail. […] Can you describe one of the situations you experienced in a university setting that you were subjected to (a situation that occurred once or a situation that was repeated over time)?

Of the 9,284 individuals who participated in the study (Bergeron et al., 2016), 3,430 reported experiencing at least one GBSVHE situation (36,9%), and 1,801 responded to the open-ended question. Some individuals described more than one GBSVHE situation in their written response, and each situation was considered separately, totaling 2,057 accounts, ranging from 5 to 950 words. Of these accounts, 81 met the selection criteria for the current study, namely: (1) the account depicted the respondent as a victim (i.e., not as a bystander or confidante), (2) it explicitly referenced the notion of consent, which was identified through a textual search using the truncated term “consen*.” Some accounts (*n* = 29) were excluded for the following reasons: they were duplicates (*n* = 11), they referred to a consent form (*n* = 2), the respondent was a bystander and not a victim (*n* = 4), or they were less than ten words in length (*n* = 4). Some individuals reported several different situations of GBSVHE in their accounts, and each incident was treated as a separate account, leading to 48 respondents for a total of 52 accounts.

### Characteristics of GBSVHE Victims

Of the sample of 48 victimized individuals, 85% identified as cisgender female, 14,5% as cisgender male, and less than 1% identified as another gender. Regarding sexual orientation, 77% of individuals identified as heterosexual and 23% as sexual minorities or in questioning. In terms of their status within the university, 52% were undergraduate students, 41% were graduate students, and 7% were professors or lecturers or employees of other professional groups. In terms of age, 60% were 18–25 years old, 31% were 26–35 years old, and 9% were 36 years old or older.

### Qualitative Analysis of the Accounts

Given the quantity and nature of the data, we adopted a qualitative descriptive approach. This method aims to describe situations and their meaning in the context in which they occurred, as close as possible to the original meaning of the words (Sandelowski, 2000). The analysis was conducted in several steps. First, major themes were identified from the description of the reported situation. Then, a situational analysis of the accounts was carried out systematically and methodically, following the guidelines of Paillé and Mucchielli (2021), which provide a framework for examining social situations, interactions, and contexts in detail. This allowed us to examine various spaces where GBSVHE occurred, the language used to describe them, the actions taken by the people involved, as well as the reaction to the event. Examining similarities and differences between the situations and how the notion of consent is articulated led us to summarize the data into two main categories of accounts: GBSVHE involving students and GBSVHE involving individuals in positions of authority. The first category includes incidents where both the victim and the perpetrator are students (undergraduate or graduate students), and the second category describes incidents where the perpetrator is in a position of authority over the victim.

The analyses were conducted in collaboration with a working committee composed of 12 individuals, including university researchers specializing in sexual violence as well as experts from different practice settings in sexual violence prevention and intervention (e.g., Sexual Assault Prevention and Aid Centres, Center on Workplace Harassment, 2SLGBTQ + Advocacy Centre). The goal of this important step was to promote multiple perspectives and facilitate a consensus regarding the interpretation of the data, in line with the tenets of collaborative research.

## Results

The results presented below are a summary of the themes identified from the experiences of GBSVHE reported in the 52 accounts. We first present recurring themes in accounts that describe situations involving students, followed by accounts of situations that involved individuals in positions of authority over the victims.

### GBSVHE Involving Students

Most of the accounts (*n* = 39) involve situations of GBSVHE among students (undergraduate and graduate students). These incidents occurred in various locations within the university (e.g., bar or student café, student union offices) or during different events of university life (e.g., student parties).

#### Coercion and Blurred Consent in Party Contexts

The first theme identified in the accounts involving students was “coercion and blurred consent in party contexts” (*n* = 22). In several universities, the introduction to campus life often takes the form of parties or events (e.g., pub crawls, “frosh week”), usually organized by student associations during the initial weeks of the academic year. These events allow students to socialize with their peers and are often characterized by heavy alcohol consumption and the normalization of sexual gestures. Within this context, respondents report facing coercive, uncomfortable, and “unhealthy” situations that undermine their ability to give genuine consent, as described in the following account:

I was pressured to show my breasts to the whole bar, my shirt was pulled, lots of guys touched my buttocks or breasts, force me to kiss. […] points were given for doing sexual acts, I kissed and let myself be touched by two guys […] it's really the whole fucking context of orientations that's unhealthy at the core. (Undergraduate student)

This account describes activities that encouraged sexual acts, with or without consent. The student conveys the coercion and pressure exerted by the party context, which blurs the line between personal desires and expected behaviors. Orientation activities typically have a challenging nature, pushing participants to go beyond their usual boundaries. When these challenges involve sexual acts, “success” is often measured by exceeding personal limits. In this context, performing sexual acts is encouraged by the unspoken rules structuring these events, promoting an “anything goes” attitude without regard for consent. This is at the expense of participants’ respect and safety. Another student's account reveals the lack of safety during an orientation activity held outside the university.

[It] happened at my undergraduate initiation night. Early in the evening, we started drinking and I agreed to kiss a guy, because yes, I wanted to. Later in the evening, this person became more insistent. […]. I first went to see a [host], who told me that “if we stopped everyone who was making out, the initiations would be boring.” I didn't expect that answer. I don't expect them to separate those who have consensual contact, we are adults after all. But the situation was different, I was telling him that I felt unsafe and that I wanted the other student to stop harassing me. […]. (Undergraduate student)

One notable finding among the accounts describing “coercion and blurred consent in party contexts” was the normalization of sexual misconduct. In various social settings within student life (e.g., evenings at the bar), the question of consent was frequently ignored, and sexual gestures were expected, with no distinction between consensual and nonconsensual acts. Heavy alcohol consumption exacerbated the issue, as described in the following account of a sexual assault:

It was our first real university party; we wanted to enjoy it. A good portion of our cohort was there. At one point in the evening, I felt very tired. I went to rest on a couch next to the dance floor. I must have fallen asleep or passed out. [Details of a sexual assault at the bar]. Of course everyone saw. Of course nobody thought I was unconscious. Even my friends just thought I was very willing. He had a blast telling his friends that I gave him a blowjob on the couch at the bar. I acted like I didn't mind (it's easier to say that yes, you were the girl who gave the guy a blowjob while you were drunk, than to say that you didn’t consent to it at all). (Undergraduate student)

This account highlights the presumption of consent and lack of intervention in situations where individuals are unable to consent. Among the accounts describing “coercion and blurred consent in party contexts,” students’ consent was often assumed without being questioned, even in cases where they were clearly incapacitated. This student's account also refers to the presence of multiple witnesses with no intervention. It reveals a failure to recognize an inability to consent and a lack of acknowledgment of the sexually abusive situation by peers.

#### Betrayal of Trust in Safe Spaces

The second theme identified in the accounts involving students was the betrayal of trust in safe spaces (*n* = 9). These accounts describe GBSVHE within university spaces perceived as safe, such as among groups and individuals who are expected to be aware of and committed to addressing GBSVHE issues (e.g., student associations or departments that stated they were committed to addressing GBSVHE). The unexpected occurrence of GBSVHE behaviors by those who are supposed to uphold safety and awareness highlights the shock and astonishment of victims. The following account describes an event involving the sexual harassment of students from the same department, illustrating the astonishment and the shock of the victim at the complacency and failure of bystanders:

I happened to go to a birthday party outside the university, but where the invited guests were exclusively fellow students of the department. We were at the restaurant, the person next to me spent the evening making sexual remarks to me, I clearly understood that the person wanted to sleep with me. It was a very intense (one-way) flirtation. Note that I had NEVER seen this person before, and this was our first interaction ever and the derogatory/inappropriate remarks started within the first 5 min of me sitting at the table! I found him pretty unbearable, by the end I found myself ignoring everything he said, pretending not to hear him and not responding. I was surprised that this person was so popular in the department that claims to be egalitarian and anti-oppressive and supposedly sensitive to issues of sexism. (Graduate student)

Other accounts like this student's reflected on their sense of trust toward the environments they were in and the groups they associated with. Despite being in spaces advocating for respect and equality, students experienced situations where these values were not upheld. The disillusionment with institutional values was especially pronounced when incidents involved other students in prominent positions within their associations.

#### Compromised Consent in Close Relationships

The third theme identified in the analyses was “compromised consent in close relationships,” which highlights the issue of consent communication in intimate relationships. Several respondents described coercive behaviors from individuals with whom they have a close relationship (e.g., classmate, dorm neighbor, peers from student involvement) (*n* = 8). They described situations in which they had repeatedly refused requests for intimate or sexual relationships, but the individual persisted. In these accounts, the interpretation of the situation revolves around the communication of consent.

The first situation involves a misunderstanding with a fellow student and friend who is also a member of the same student association. We quickly became close, and this led to several misunderstandings about what certain gestures on my part meant, especially in a drunken situation. I dodged his approaches several times (attempts to kiss me, or intrusive gestures) and clarified things verbally afterwards when sober. […]. The last time an inappropriate proposition occurred I was not drunk and this time I am certain I did not imply anything by my verbal or non-verbal behavior. […]. The main problem in this situation, in my opinion, [is that] I have not yet managed to [express it] clearly to the person involved. (Graduate student)

What emerges here is the belief that the “no” cannot be heard or understood, even though it is clearly stated. The student concludes that she failed to express her nonconsent effectively, attributing the recurrence of unwanted gestures to a communication failure. This underscores the undue responsibility placed on women to be the gatekeepers of consent. Additionally, in situations that involved alcohol consumption, victims felt they miscommunicated their refusal due to intoxication, despite verbally expressing it.

My apartment neighbor, who was also a student, after a university happy hour tried to assault me. We were at his house, in his bed, he wanted to have sex with me, was touching me, and I said no. Several times I told him I didn't want to have sex with him, but he kept touching me, penetrating me with his fingers. I don't think he understood that I was not consenting, because of the alcohol. He would need to be educated. (Undergraduate student)

What emerges here is that “miscommunication” of nonconsent was associated with the victim's level of intoxication, whereas “misunderstanding” of nonconsent was attributed to the perpetrator's intoxication or lack of education. The relational context, including intoxication, reputation, and trust, shapes consent communication. These factors complicate the clear recognition of nonconsent, as highlighted in this student's discomfort:

In the spring of [year] I met a guy and we started dating. Throughout our relationship he was a perfect gentleman. At the end of that spring, I wanted to end our relationship, but he didn't agree with me. At some point, I got tired of constantly explaining to him why I wanted to break up and started ignoring his calls and messages. […]. I quickly made it clear that I had no interest in getting back together with him and that I considered him a friend. Since then, he frequently invites me for a drink, stares at me when we are both in the cafeteria […]. At social events where alcohol is involved, he tends to stick by me, tries grabbing me by the waist or pressing himself against me (in other words, touching me in what I consider to be an inappropriate way). And if a guy, other than my guy friends, starts a conversation with me, he inevitably comes between us. I know he is a good person and would never go too far, that in his head he is just flirting. To a certain extent I trust him. But his actions and words make me uncomfortable. (Graduate student)

Additionally, a significant finding in this category of accounts was that coercive behaviors (e.g., insistence, harassment) were described but not acknowledged as such. In some cases, as described in the following account, communication, understanding of consent, and trust can be manipulated by the perpetrator:

He made me feel like I wanted this to happen, that I was in some kind of relationship with him. I don't understand what's happening to me, I believe him, I think I'm the one who wanted all this, so I agree to see him again. This was followed by two evenings of full sexual relations where I “consented” without really understanding what was happening to me. Then I realized, alone at home, that it was a sexual assault and that he had taken advantage of my guilt and my fear of displeasing him to manipulate me. From that moment on, I refused to speak to him again and I ignore him when I see him at the university. I am ashamed that I fell for it and agreed to have sex with him, so I haven't told anyone. (Graduate student)

This student stated that she gave her “consent” without truly understanding it because she did not want the sexual relationship. She questions her own judgment and expresses a sense of shame at being “manipulated,” which leads her to remain silent.

This first section presented themes identified in accounts describing GBSVHE involving undergraduate and graduate students. Although certain university settings (e.g., orientation activities, party context) see more frequent occurrences, GBSVHE also occurs in spaces with individuals aware of gender-based and sexual violence issues. A subset of accounts specifically described coercion in close relationships among students and highlighted the aspect of communication and understanding of consent. In each of these spaces, intoxication, insistence, and coercion were frequently not recognized as invalidating consent, leaving victims feeling responsible and silent about their experiences.

### GBSVHE Involving Individuals in Positions of Authority

The second category of accounts (*n* = 13) involved students and employees reporting incidents perpetrated by individuals in a position of authority, including professors/lecturers (*n* = 7), thesis advisors (*n* = 5), and sports coaches (*n* = 1). These accounts described situations that evolved gradually and GBSVHE events that occurred multiple times:

[The professor] seduced me and used his reputation as a professor…. (married man) … paid for food and booze, etc. and then lured me back to the hotel… I was uncomfortable with his little game, I knew he was clearly taking advantage of the situation, but hey… I was 20 years old… and did not really have a concept of free and informed consent at the time… (Undergraduate student)

I had sex with a professor in my third year of university. I wanted it, but I was also kind of in admiration with him; he seemed so exceptional. He asked me to come to his house. I refused. […] He always wanted to buy me beers, take me out to eat, etc. I went to his house two or three times. I was uncomfortable during the act, I felt disconnected, I found it a bit disgusting, but at the same time, I had consented. I did everything I could to not run into him again, he wrote me poems and also a message to tell me that he was a good person, that if I had heard rumors about him, they were false. I ended the relationship, and I never responded to his emails again. This is not so much an assault as an abuse of power. (Undergraduate student)

These accounts illustrate how individuals abuse the power granted by their status (e.g., reputation, charisma) to make repeated advances and limit the possibilities of rejection. The first notable finding in this second category of accounts was the abuse of power that creates an environment where students and university employees do not feel free to refuse.

The main situation occurred while my master's advisor and I were out of town together. We were sleeping in the same hotel room. […]. That night he offered me to join him in his bed and I accepted even though I didn't feel like it and was uncomfortable. He didn't force me or threaten me in any way; however, I didn't really feel free to refuse, I felt like it I couldn’t do that. The next day he gave me money, officially to compensate me for my participation in the training; but I mostly felt like he was buying my silence. (Graduate student)

The abuse of power often involved exploiting the trust and dependency of students, making it difficult for them to refuse or recognize the coercion. Respondents also elaborated on the emotional and psychological impact and described feeling frozen, disgusted, and uneasy. The imposed conditions led to a sense of constraint on their freedom to refuse sexual relationships and a sense of powerlessness among the victims.

With hindsight, however, I really consider that he did not have to act that way; he was clearly in a position of authority (director and immediate superior since I was also his teaching assistant; he was the one who provided me with work, I “depended” on him in many ways) and I think that he took advantage of the circumstances (isolation, distance from familiar settings, I was coming out of a relationship, etc.) to obtain consent that was clearly not proactive. I did absolutely nothing to lead to a sexual relationship, even though I agreed to “go through” with it. (Graduate student)

The second notable finding in accounts involving individuals in positions of authority was the betrayal of trust. Respondents specifically referred to a breach of trust, where individuals in positions of authority manipulated their relationships with students or university employees. This breach of trust often led to confusion and difficulty in labeling the situation as nonconsensual, as described in the following account:

At a few parties where there were students and professors, my thesis director made inappropriate compliments to me. During a very drunken evening at my place, he made advances to me. We found ourselves alone. We kissed. I was too drunk to realize what was going on. When I came to my senses, he was more or less raping me (I say more or less because I didn't resist, but I wasn't aware of what was going on and I never wanted to have sex with him). I screamed and told him to stop and went to wash up. He tried to calm me down. I didn't know what to do or think. He spent the night at my place. This happened twice, even though this person physically disgusted me. Once, unrelated to the conversation, he insisted that we were between adults and that we shouldn't mess around with the question of whether or not we were consenting. I later realized that I preferred to make myself believe that I wanted to rather than see myself as a victim of abuse: I couldn't accept seeing myself as a victim or seeing him as an abuser. And I couldn't relate to it because I didn't want what happened, but I couldn't call it rape either because I didn't put up any physical resistance. (Graduate student)

What emerges here is the reluctance to label the situation as nonconsensual as it could affect the perception of oneself (considering oneself as a victim) and of others. Admitting to a transgression of consent implies acknowledging there was abuse. For the next respondent, the unequal power dynamic affects the way she perceives the relationship, creating significant discomfort and placing her in a difficult position:

I was completely frozen. We had sex and then I went to my room. This may sound pretty far from an assault situation… I was pretty willing, despite it all. The problem was that he used his position as my superior to convince me and impose conditions. I was not to talk about what happened. The next day, the following week and the years that followed were punctuated by encounters, hidden, decided by him. […]. When I wanted to have a more meaningful relationship with him, he put an end to our encounters outside the university. […] Basically, my opinion did not matter to him. He was the one who decided everything: when we would see each other… and when we would stop seeing each other. The violence came from there: there was no equality between us. I couldn't control the situation […]. I had to act like nothing was wrong, even though I was hurt, because no one in the department was supposed to suspect that we had an affair. (University employee)

For this respondent, the unequal relationship is the source of violence and constraints on her freedoms. She describes the constraints imposed by the professor, who used his position of power to “convince and impose” while she was a student. The perpetrator was initially her supervisor and later her employer, highlighting the continuation of abuse despite changes in status within the university. Another student explains how she trusted her thesis advisor despite his reputation for making gestures toward female students in the department:

I thought he saw me as asexual in some way because he once told me, “Don't worry, I don't fuck the payroll.” In addition, a colleague (another professor in the department and a very good friend) had also told me that he never “touched” his female students, the ones he supervised. Then the episode happened. It was at a party that started off well, I was having fun, had a few drinks with my colleagues and my director. Then he asked me if I wanted to go upstairs with him to “sleep with him.” I was in shock and answered with a nervous laugh, “Hahaha! Very funny.” He replied that it was not a joke. I told him that I was not interested at all. He asked me why, holding me tighter in his arms. Trying to pull myself out of the embrace and realizing that I would have to justify my refusal, I adopted a diplomatic tone to remind him that he was my director, and I was his student. He answered back with a “Yeah, so?” I then reminded him that I was working on male/female power relationships. He cut me off and said, “Stop arguing! Shut up, bitch!” From that moment on, everything is a blur… I don't remember what I said back, only that he said “Stop arguing! Shut up bitch!” He held me even tighter against him, his hands moving from my back to my buttocks as I tried to untangle myself. I remember looking around and seeing some of the other professors looking at us funny and talking amongst themselves, but no one intervened? (Graduate student)

As described above, some incidents occurred in public spaces, such as parties with students and professors, where nonconsensual acts were visible to others but went unchallenged. This lack of intervention further underscores the abuse of power and the normalization of such behaviors within certain university settings.

This second section outlined situations of GBSVHE involving individuals in a position of power over the victim. In the context of educational or authority dynamics, consent is compromised, as it is impossible to ensure that it is free and informed. The individuals involved (professors, thesis directors, coaches, employers) leveraged their power and authority to influence how the situation is perceived (“we are consenting adults”). Respondents found it difficult to label their experiences as sexual violence. Many required time and distance to acknowledge the abuse of power and the betrayal of trust.

## Discussion

This article examined the notion of sexual consent as articulated in the written accounts of individuals who were victims of GBSVHE. Respondents were asked to describe a situation of sexual violence in a university setting as part of a larger quantitative study (Bergeron et al., 2016) and spontaneously referred to the word “consent” in their accounts. The analyses explored the notion of consent in GBSVHE involving perpetrators who were other students (undergraduates or graduate students) and GBSVHE involving individuals in a position of authority (e.g., professor, supervisor) over the victim (students or university employees). Respondents described their internal state of unwillingness in various contexts, the communication of their refusal, the reaction of others (perpetrator, bystanders), and their interpretation of the situation. Several themes emerged from the accounts: coercion and compromised consent in social events (e.g., orientation activities, student parties), normalization of sexual misconduct, the presumption of consent, lack of intervention by bystanders, betrayal of trust, disillusionment with institutional values, miscommunication or misunderstanding of consent, coercion by trusted individuals, the influence of alcohol and social context, abuse of power, emotional and psychological impact, and difficulty in labeling the situation as nonconsensual.

Overall, the results indicate that the notion of consent is far more complex than a simple “yes” or “no,” aligning with previous research (Beres, 2010; Fenner, 2017; Muehlenhard et al., 2016; Savoie et al., 2019). Various contextual elements (e.g., locations, events, relationship to the other individual) influence the ability to consent and the freedom to affirm or withdraw it. In GBSVHE situations, communication of nonconsent often goes unheard and transgressed, and the inability to consent is frequently overlooked, even by witnesses. Whether perpetrated by another student or by an individual in a position of authority, the situations reveal coercion by trusted individuals, normalization of sexual violence, psychological impact, influence of social and institutional norms, and betrayal of trust.

## Difficulty in Labeling Situations as Nonconsensual

What is striking in these accounts is the discrepancy between the description of the internal state of unwillingness and how consent is articulated in the written answers. Respondents often used ambivalent terms such as, “I was half-consenting,” “yes, a sexual act was committed without my consent, but I was far from being assaulted,” or “he had a sexual relationship that was half-consented to.” This discrepancy underscores the complexity of consent, where individuals struggle to clearly label their experiences as nonconsensual. These results provide additional support to previous calls to integrate nuances of consent beyond verbal affirmation (Beres, 2014; Setty, 2023).

Additionally, the results reflect the intricacies between consent and sexual violence, revealing difficulties (and sometimes reluctance) in characterizing the situation as sexual violence and recognizing invalid consent. As reported in their accounts, victims often blamed themselves for failing to communicate their refusal properly and attributed the recurrence of coercive behaviors to a lack of understanding on the perpetrator's part. Here, overemphasizing consent communication can inadvertently perpetuate victim blaming. When the focus is solely on how well the refusal was communicated, it can lead to blaming victims for not being clear enough, ignoring the perpetrator's responsibility. Furthermore, as reported in previous work (Beres, 2010; Hansen et al., 2010), certain situations reflect not a lack of understanding but a deliberate intention to transgress the boundaries of the other individual, notably by using manipulation or verbal abuse.

The discrepancy in the understanding and articulation of consent as well as the difficulty in labeling situations as nonconsensual, reflect broader issues within rape culture and institutional norms. Rape culture, characterized by the normalization and trivialization of sexual violence, perpetuates myths that blame victims and excuse perpetrators (Ricci & Bergeron, 2019), reinforcing ambiguity in identifying and labeling nonconsensual experiences. As observed by Dardis et al. (2021), victims of sexual assault often interpret their experience as miscommunication due to a discrepancy between the actual incident and their internalized notion of what constitutes a sexual assault. In the context of university settings and institutional norms specifically, acknowledging nonconsent requires a significant re-evaluation. This process involves recognizing oneself as a victim, which can be difficult due to internalized beliefs about personal responsibility and the desire to avoid the victim label (Dardis et al., 2021). Additionally, it challenges the perception of the university environment as a safe space, forcing individuals to confront the reality of institutional failures in protecting students. Furthermore, it entails reevaluating the trust placed in the perpetrator, who is often seen as a trustworthy peer or authority figure, complicating the recognition of their actions as aggressive.

### Unequal Power Dynamics

Power dynamics significantly hindered the acknowledgment and negotiation of nonconsent in university settings, especially through gendered dynamics, social norms, and authority relationships. As noted in previous studies (Savoie et al., 2019; Willis et al., 2019), our results reflect the weight of heteronormative norms and sexual stereotypes that position women as the gatekeepers of sexual consent and men as the initiators who may persist in obtaining it. These norms place an undue burden on women to regulate sexual encounters, while men are often socially encouraged to persist despite resistance. This dynamic is exacerbated by social norms that tolerate, minimize, or justify sexual violence, thereby perpetuating rape culture, as evidenced in accounts describing blurred or presumed consent during social events. Existing research indicates that the framing of consent is heavily influenced by rape culture, particularly in situations involving significant alcohol consumption (Dardis et al., 2021; Pugh & Becker, 2018). This context often obscures the boundaries of consent, making it difficult to recognize and address sexual violence effectively, as supported in accounts describing the normalization of sexual misconduct.

Furthermore, unequal gender norms are intertwined with institutional power structures. Social norms and unequal power dynamics are deeply interconnected, particularly in the context of consent and sexual violence (Hirsch & Khan, 2020). Unequal power dynamics are first apparent among students in incidents involving individuals who occupy a prominent position (e.g., in a student association) and leverage their influence to coerce or manipulate consent. Unequal power dynamics are most evident in the authority relationships within the university setting, where individuals in positions of power (e.g., professors, supervisors) exploit their power, making it difficult for those with less power to refuse or acknowledge nonconsensual situations. The accounts illustrate how individuals in authority exploit these dynamics to exert control, highlighting the intersection of institutional authority and gendered power imbalances. Academic and authority relationships invalidate consent due to the inherent power imbalance in these relationships. Holding a higher status within the university does not necessarily invalidate consent, rather, it is within the unequal dynamic that consent is compromised. When one party holds a significant influence or authority over another, such as a president of a students’ association over a first-year undergraduate or a supervisor over an employee, the subordinate may feel pressured to comply with requests and demands, including sexual ones, due to fear of negative consequences or hopes of favorable outcomes. Consequently, these dynamics perpetuate environments where consent is not freely given and sexual violence is normalized or overlooked.

### Unsafe Conditions

Universities have a responsibility to ensure a climate of safety and trust. Every member of the university community, whether student or staff, has the right to expect an educational and professional environment that is free of all forms of gender-based and sexual violence. In several accounts, expectations of safety toward the institution and its individuals were crucial in recognizing coercion and GBSVHE. Expectations of safety, coupled with the generally held view that academic environments are inherently safe, may hinder the recognition of GBSVHE. Department or student union parties, for instance, were expected to be violence-free environments. The betrayal of trust in these ostensibly safe spaces exacerbated the impact of GBSVHE, fostering disillusionment with the institution's purported values of respect and equality.

Moreover, several incidents occurred in public spaces, such as parties with students and professors, where nonconsensual acts were visible to others but went unchallenged. The lack of intervention in instances of violence—whether by peers or employees—further erodes trust and reinforces the perception that the institution tolerates such behavior (e.g., refusal to intervene by students who organize social events, failure to intervene when faced with a GBSVHE situation, keeping individuals who have committed acts of GBSVHE). This failure to act not only perpetuates unsafe conditions but also undermines the credibility and integrity of the institution.

Regarding the accounts describing GBSVHE involving individuals in positions of authority specifically, patterns of inaction suggest the presence of a protective system that prioritizes maintaining the status quo and reputation over addressing safety and justice. This protective system may indicate a systemic issue within academia and warrants further investigation. Feminists and student movements have increasingly criticized university administrations for perpetuating or concealing such violence through inaction (Ricci & Bergeron, 2019). Exploitation of power in the university setting is often facilitated by institutional practices and norms that tolerate or minimize such behaviors, reinforcing the systemic nature of gender-based and sexual violence in academic environments. These intertwined dynamics highlight the need for institutional reforms to address and dismantle the perpetuation of rape culture and ensure genuine consent.

### Strengths and Limitations

This study has several strengths and limitations. First, the study extensively explored issues surrounding sexual consent and sexual violence within the university setting, addressing existing gaps in the literature. By including the entire university population (e.g., graduate students, employees), the study led to the identification of similar experiences regardless of one's status within the university (e.g., normalization of sexual misconduct, betrayal, lack of intervention). Additionally, relying on the spontaneous use of the word “consent” allowed for an exploration of how respondents articulated this notion without being influenced by the researchers’ questions or vocabulary.

There are, however, some methodological limitations that arise from the nature of the data. The accounts were numerous and of varying length, reflecting what was deemed significant and meaningful to the individuals who wrote them at the time of their recounting. Consequently, it is possible that some aspects of their experiences were incomplete or have not been mentioned. Additionally, the accounts were collected in 2016, before significant social changes such as #MeToo in 2017 and the implementation of mandatory training and prevention strategies in Québec, Canada. As a result, the perspectives and experiences may not fully capture the current understanding and attitudes toward these issues. Nevertheless, these insights can serve to inform current training programs and serve as a starting point to explore changes in the experiences of GBSVHE over time. The descriptive approach allowed for a comprehensive exploration of consent, uncovering the complex interplay of power dynamics and revealing dimensions that have received little attention in previous research (e.g., contexts involving individuals in positions of authority).

### Implications and Recommendations

The realities revealed in this study underscore several critical implications and recommendations for addressing gender-based and sexual violence in higher education. First, the study highlights the need for a more nuanced understanding of consent beyond verbal affirmations. Restricting the notion of sexual consent to its communicative dimension risks perpetuating rape myths and victim-blaming, placing undue blame on victims who have “failed to properly communicate.” Additionally, the influence of unequal power dynamics and gendered and social norms need to be addressed when discussing consent and sexual violence. Consent education, including affirmative consent and grey area trainings, remains important (Setty, 2023) and should incorporate challenges to social norms (e.g., heteronormative standards, gender-based power imbalances such as expectations of male assertiveness and female compliance) and support the identification of conditions that render consent invalid (e.g., intoxication, coercion, position of authority). The normalization of misconduct in supposedly safe environments and the issue of institutional betrayal highlight the need for institutional change and courage. Universities must promote cultural change, challenge social norms, and ensure transparency and accountability to rebuild trust. As advocated by other scholars (Beres, 2022; Hirsch & Khan, 2020), this study advocates for upholding ethical conduct and respectful, egalitarian relationships to ensure personal freedom.

Future research should explore the long-term impact of institutional changes and consent education programs implemented recently. Additional research is needed on the influence of power dynamics, institutional betrayal, and inaction in higher education. Moreover, it is crucial to investigate how perpetrators manipulate the notion of consent to coerce, often using trust, authority, or social norms to blur the lines of genuine consent in university settings.

## Conclusion

This study explored how members of the university population (students, employees) articulated the notion of consent in their experiences of GBSVHE, revealing the difficulty in labeling situations as nonconsensual, the influence of unequal power dynamics, and institutional norms and practices. By documenting these experiences, this research contributes to a deeper understanding of GBSVHE and calls for social and institutional change. Mobilizing all members of the university community will be essential to make ethical and egalitarian relationships the norm throughout the academic career and in all structures.
